# Human and gut microbiota synergy in a metabolically active superorganism: a cardiovascular perspective

**DOI:** 10.3389/fcvm.2024.1411306

**Published:** 2024-10-11

**Authors:** Matteo Antonio Russo, Matteo Puccetti, Claudio Costantini, Stefano Giovagnoli, Maurizio Ricci, Enrico Garaci, Luigina Romani

**Affiliations:** ^1^ San Raffaele IRCSS, Rome, Italy; ^2^ Department of Pharmaceutical Sciences, University of Perugia, Perugia, Italy; ^3^ Department of Medicine and Surgery, University of Perugia, Perugia, Italy; ^4^ San Raffaele Research Center, Sulmona, L’Aquila, Italy

**Keywords:** cardioimmunology, cardiovascular diseases, tryptophan, indole-3-aldehyde, Aryl hydrocarbon receptor

## Abstract

Despite significant advances in diagnosis and treatment over recent decades, cardiovascular disease (CVD) remains one of the leading causes of morbidity and mortality in Western countries. This persistent burden is partly due to the incomplete understanding of fundamental pathogenic mechanisms, which limits the effectiveness of current therapeutic interventions. In this context, recent evidence highlights the pivotal role of immuno-inflammatory activation by the gut microbiome in influencing cardiovascular disorders, potentially opening new therapeutic avenues. Indeed, while atherosclerosis has been established as a chronic inflammatory disease of the arterial wall, accumulating data suggest that immune system regulation and anti-inflammatory pathways mediated by gut microbiota metabolites play a crucial role in a range of CVDs, including heart failure, pericardial disease, arrhythmias, and cardiomyopathies. Of particular interest is the emerging understanding of how tryptophan metabolism—by both host and microbiota—converges on the Aryl hydrocarbon Receptor (AhR), a key regulator of immune homeostasis. This review seeks to enhance our understanding of the role of the immune system and inflammation in CVD, with a focus on how gut microbiome-derived tryptophan metabolites, such as indoles and their derivatives, contribute to cardioimmunopathology. By exploring these mechanisms, we aim to facilitate the development of novel, microbiome-centered strategies for combating CVD.

## 1 Introduction

Cardioimmunology is a field of study that focuses on the interactions between the cardiovascular system and the immune system (1–5). It explores how the immune system can impact various cardiovascular diseases, such as atherosclerosis, myocardial infarction, cardiomyopathies, arrhythmias and heart failure. Research in cardioimmunology aims to understand the complex mechanisms by which immune cells and molecules contribute to both the pathogenesis and resolution of cardiovascular diseases (CVD) (6). By uncovering these interactions, scientists hope to develop new strategies for preventing and treating cardiovascular conditions through targeted immunomodulatory therapies (7).

Cardioimmunology, indeed, is a relatively new and rapidly growing field that focuses on the intersection of CVD and the immune system (8). It explores the complex interactions between the immune system and the cardiovascular system, as well as the role of inflammation in the development and progression of various cardiovascular conditions (9, 10). In the last decade, the systemic dimension has emerged as a predictor of CVD, alongside cardiomyopathies and arrhythmias. Immunomodulation in the local microenvironment, metabolism and mitochondria of the cardiac tissue are highly responsive to the environment (10–15). Experiments involving factors such as gut microbial composition (e.g., considering the host-microbiome dyad as a “superorganism”), the circadian clock (16) or hypoxia (17) have been shown to impact tissue function, leading to conditions such as cardiometabolic disorders, myocarditis, arrhythmias along with tissue remodeling notably with occurrence of fibrosis. Similarly, systemic inflammation, immune cells and oxidative stress also contribute to the pathogenesis of hypertension, which is a significant risk factor for CVD, through vascular inflammation and microvascular remodeling (18).

Mapping cell destinies, depleting and regenerating immune cells in experimental models of heart disease, along with analyzing the human heart at a cellular level, significantly enhance our comprehension of the intricate communication between immune and non-immune cells within the heart (19, 20). Although the immediate immune reaction is crucial for triggering inflammation and repairing tissue after damage, prolonged activation leads to harmful effects, influencing negative changes in the heart’s structure and function. Identifying the precise roles of immune cells within the cardiac setting opens up novel avenues for adjusting immune responses to manage inflammation effectively in cases of heart diseases.

## 2 Key aspects of cardioimmunology

**Figure 1:**
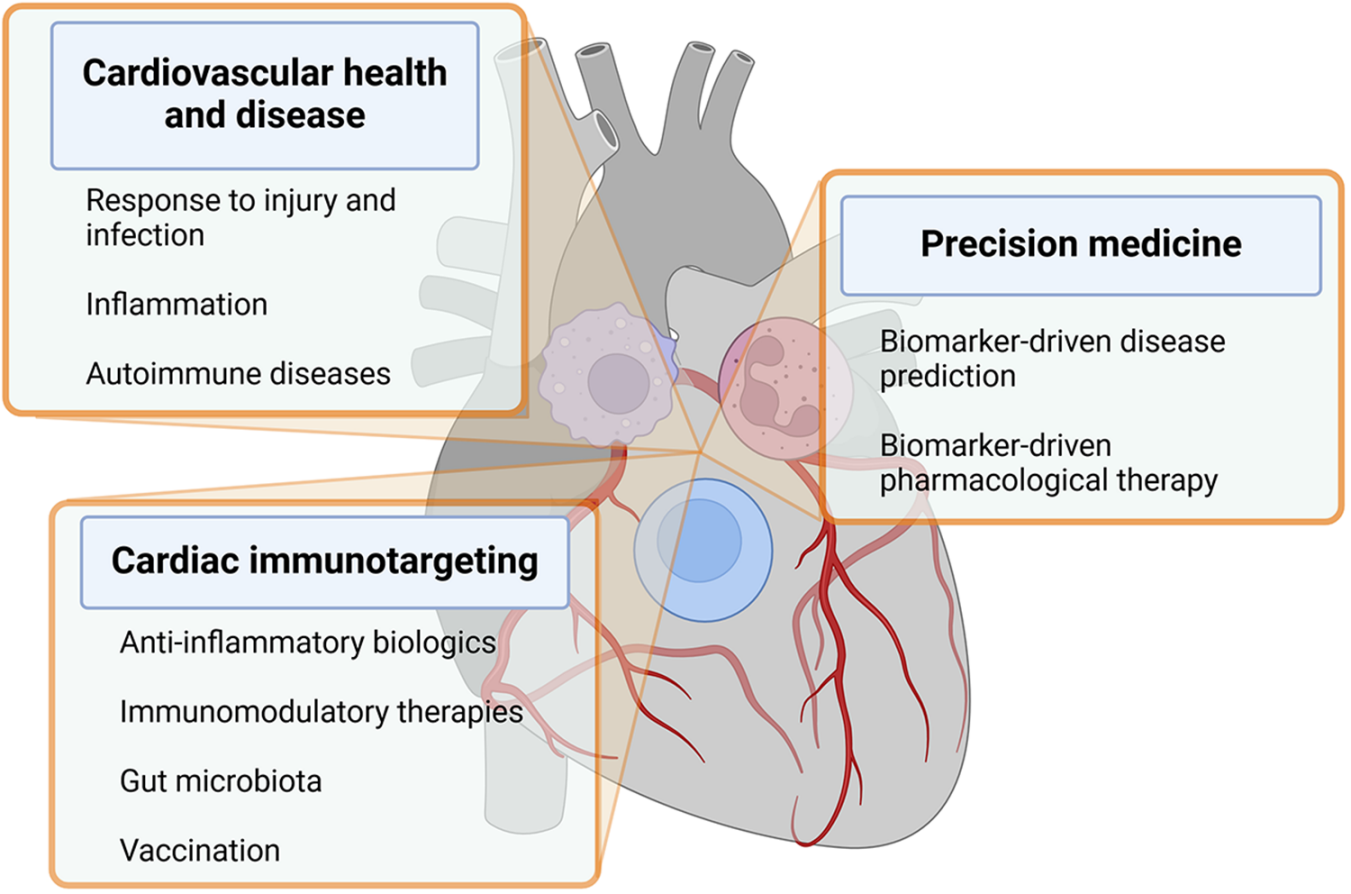
The figure recapitulates key aspects of cardioimmunology within text boxes departing from immune cells overlying a human heart. Details are described in the text. Created with BioRender.com.

The heart incorporates immune cells as crucial cellular elements that engage in communication with resident cardiac cells in situations of homeostasis, cardiac injury, and remodeling (21, 22). These discoveries play a pivotal role in shaping and broadening the emerging domain of cardioimmunology. In this analysis, we examine the latest literature related to this subject and deliberate on the ongoing and prospective initiatives aimed at propelling this field ahead. Some of the crucial aspects of cardioimmunology are depicted in Figure 1 and listed below.

(a) *Inflammation and cardiovascular disease*: Chronic inflammation is now recognized as a key driver of CVD, including atherosclerosis, cardiomyopathies, arrhythmias, heart failure, and myocarditis. Immune cells and inflammatory mediators play a crucial role in the development of these conditions (12, 23).
(b) *Immune cells in cardiovascular health and disease*: Immune cells such as macrophages, T cells, and neutrophils play important roles in maintaining cardiovascular health and responding to injury or infection in the heart and blood vessels (22).
(c) *Immunomodulatory therapies for CVD*: Researchers are exploring the potential of targeting the immune system to develop novel therapies for CVD (24). This includes investigating the use of anti-inflammatory agents, immune-modulating drugs, and biologics to reduce inflammation and improve outcomes in patients with heart disease (25–27).
(d) *Biomarkers of inflammation in CVD*: Biomarkers of inflammation, such as C-reactive protein and IL-6, are used to assess the level of inflammation in patients with CVD. Monitoring these biomarkers can help guide treatment decisions and predict outcomes (28).
(e) *Cardiovascular complications of autoimmune diseases*: Some autoimmune diseases, such as rheumatoid arthritis and systemic lupus erythematosus, are associated with an increased risk of cardiovascular complications due to chronic inflammation and immune system dysregulation (29).
(f) *Ongoing directions*: Current research in cardioimmunology aims to further elucidate the mechanisms underlying the crosstalk between the immune system and the cardiovascular system, identify new therapeutic targets, and develop personalized approaches to prevent and treat CVD based on immunological profiles (10). Paradigmatic in this context are the mechanistic studies linking autoimmunity and inflammation to the development of arrhythmia (Box 1).

Box 1 **The role of immunity and inflammation in arrhythmia development.**

Arrhythmias associated with autoimmunity and inflammation occur through at least four mechanisms that disrupt electrical coupling:

**1. Autoimmunity targeting ion channels and junctional molecules of the intercalated disc and lateral gap junctions.** The clinical manifestation involves the presence of autoantibodies against ion channels (Na+, Ca2+, K+) and junctional molecules (connexins and intercalated disc molecules). It has been proposed that these arrhythmias may respond to mild immunosuppressive treatment, which suppresses autoantibody production (30).
**2. Direct effects of proinflammatory mediators and cytokines on channel function and gap junction coupling.** Molecules like histamine and prostaglandins, which increase cytosolic c-AMP/c-GMP, temporarily disrupt cell-to-cell junctions, leading to electrical uncoupling between cardiomyocytes at the intercalated disc level (31). Cytokines such as TNF-α, IL-1β, and IL-6 can also affect both cardiomyocyte channels and gap junctions, thus influencing electrical activity and leading to cytokine-associated arrhythmias (32).
**3. Immuno-mediated damage of excitable and conduction tissue,** resulting in blocks, bradycardias, and various types of tachyarrhythmia (33–35). Myocardial cell death and myocardiosclerosis following immuno-inflammation are frequent arrhythmic causes in myocarditis. Also, these arrhythmias may respond to mild immunosuppressive treatment, when allowing the repair of sublethally damaged tissue.
**4. Arrhythmogenic cardiomyopathy**, a rare heart disease characterized by structural and electrical alterations, including abnormalities in intercalated discs and fibro-fatty replacement of ventricular myocardium. While familial genetic factors play a significant role in its pathogenesis, subsequent alterations in intercalated disc junctional complexes contribute to the clinical presentation, especially involving desmosome and tight junction components. Autoantibodies against tight junction and desmosome-associated molecular components have been described (36).

Overall, cardioimmunology represents an exciting and promising area of research that has the potential to improve our understanding of CVD and lead to the development of innovative therapies to combat these conditions. In particular, some emerging areas of particular interest are represented by:

(i) The role of the gut microbiota and products thereof. The gut microbiota plays a pivotal role in educating and modulating the immune system, influencing the balance between pro-inflammatory and anti-inflammatory responses. On the one hand, dysbiosis of the gut microbiota, characterized by alterations in microbial composition and function, has been linked to immune dysregulation and chronic low-grade inflammation, which are key drivers of atherosclerosis, hypertension, and other cardiovascular conditions. Understanding the interconnections between the cardiovascular and the gut microbiota opens avenues for developing novel therapeutic strategies (37, 38). On the other hand, the intricate crosstalk between the gut microbiota and the cardiovascular system underscores the importance of considering the gut as a key player in cardioimmunology (39). By elucidating the mechanisms by which the gut microbiota influences immune responses, inflammation, and metabolism in the context of cardiovascular health, researchers may uncover novel therapeutic targets and strategies for the prevention and management of several pathologic conditions, including CVD (39, 40).
(ii) The potential role of anti-inflammatory biologics in CVD. The gut microbiota produces a myriad of bioactive metabolites and signaling molecules that can impact immune function and cardiovascular health. Short-chain fatty acids (SCFAs), produced through the fermentation of dietary fibers by gut bacteria, have been shown to exert immunomodulatory effects by regulating the differentiation and activity of immune cells, thereby influencing inflammation in the cardiovascular system (41). Beyond SCFAs, the gut microbiota generates a diverse array of metabolites, including trimethylamine N-oxide (TMAO), lipopolysaccharides (LPS), and bile acids (BAs), which have been implicated in the pathogenesis of CVD (42). On the one hand, the exploration of anti-inflammatory biologics in cardiovascular diseases, particularly anti-inflammatory and immunomodulatory indole derivatives, represents an exciting avenue for research and therapeutic development (43). While there is evidence suggesting their potential benefits, ongoing studies and further clinical trials are necessary to establish their role in specific patient populations and to refine treatment strategies. On the other hand, chronic inflammation is a hallmark of many cardiovascular diseases, and the gut microbiota has been implicated in the regulation of inflammatory pathways that contribute to vascular dysfunction and atherogenesis. Through the activation of toll-like receptors and nucleotide-binding oligomerization domain-like receptors, gut-derived microbial products can trigger inflammatory cascades that promote the development of cardiovascular pathology. Some biologics that target inflammatory pathways, such as TNF-α inhibitors or IL-1 inhibitors (26, 27, 44), may have indirect heart-protective effects by reducing systemic inflammation, which is a risk factor for cardiovascular disease (45). Overall, targeting the gut microbiota represents a promising avenue for the development of novel therapeutic strategies in cardioimmunology. Approaches such as probiotics, prebiotics, postbiotics, and fecal microbiota transplantation offer the potential to modulate the gut microbiota composition and activity, thereby influencing immune responses and mitigating cardiovascular risk factors (46–48).
(iii) Vaccination and cardiovascular health. Some research in cardioimmunology explores the impact of vaccinations on cardiovascular health. For instance, vaccines targeting infectious agents may not only prevent infections but also impact the risk of cardiovascular events associated with inflammation. In particular, vaccination plays a crucial role in maintaining overall health, including cardiovascular health. While vaccines primarily target specific infections, their impact can extend beyond preventing the targeted diseases. Here are some aspects of vaccination and cardiovascular health (49). A prototypic example is represented by influenza vaccine: Influenza infections can lead to respiratory complications, and severe cases may have cardiovascular implications. By preventing the flu, the vaccine helps reduce the risk of influenza-related cardiovascular events. Not secondarily, COVID-19, caused by the SARS-CoV-2 virus, has been associated with various cardiovascular complications. These include myocarditis, pericarditis, and an increased risk of blood clot formation. COVID-19 vaccination has been shown to be effective in preventing severe illness and complications, including those related to the cardiovascular system (50). Nevertheless, the very vaccine, owing to its formulation (i.e., mRNA in lipid nanoparticles) can exert direct effects on the cardiovascular system (51, 52). Relevant in this regard are experimental studies on autoimmune myocarditis. Box 2 provides some information in this regard.

Box 2 **Autoimmune myocarditis in viral infection.**

Autoimmune myocarditis is often indirectly linked to viral infections (53). One potential contributing factor is the release or exposure of cardiac myosin following viral-mediated myocyte damage, triggering autoimmune responses and myocardial inflammation (54, 55). Fulminant myocarditis spontaneously develops in Pdcd1^–/–^ Ctla4^+/–^ mice. Axelrod et al. (56) conducted single-cell sequencing and TCR sequencing of immune cells infiltrating these myocarditis tissues, revealing a significant increase in the number of CD8^+^ T cells with clonal expansions. The removal of CD8^+^ T cells prevented myocarditis development, while the removal of CD4^+^ T cells did not alter myocarditis incidences. Adoptively transferring CD8^+^ T cells from Pdcd1^–/–^ Ctla4^+/–^ mice to Rag1^–/–^ mice led to myocarditis development after two months, emphasizing the pivotal role of CD8^+^ T cells in fulminant myocarditis (56). Additionally, a recent study highlighted macrophage migration as a notable histopathological feature of myocarditis, suggesting that targeting macrophages could be a potential therapeutic approach for this disease (57). Treating mice with a nontoxic endogenous Aryl hydrocarbon Receptor (AhR) ligand, ITE [2-(1'H-indole-3'-carbonyl)-thiazole-4-carboxylic acid methyl ester] ameliorated cardiac function. It was hypothesized that activation of AhR by ITE in myocardial infarction mice would boost regulatory T-cell differentiation, modulate macrophage activity, and facilitate infarct healing (58).

## 3 Human and gut microbiota synergy on tryptophan utilization

Both humans and gut microbiota feed on dietary Trp for proteogenesis and other functions that rely of the degradation of this essential amino acid. The phylogenesis of Trp catabolism across different organisms provides insights into the evolutionary advantages and diverse functions of this pathway (59). Trp catabolism is a highly conserved process that has evolved over time, and its existence across various organisms suggests its importance in adaptation and survival (60–62). Here are some key points regarding the phylogenesis of Trp catabolism: (a) *Universal presence*: Trp catabolism is found in a wide range of organisms, including bacteria, fungi, plants, and animals. This universality indicates that the pathway has been evolutionarily conserved and is fundamental to the biology of diverse life forms (60); (b) *Metabolic regulation*: The regulation of Trp catabolism has likely evolved as a mechanism for organisms to adapt to changing environmental conditions, nutrient availability, and immune responses (63). In many cases, the regulation of Trp catabolism is responsive to external stimuli, such as stress, infection, or inflammation, highlighting its role in the adaptive response to various challenges; (c) *Host-pathogen dynamics*: The evolutionary arms race between hosts and pathogens has likely shaped the development and diversification of Trp catabolism. Hosts may have evolved this pathway as a defense mechanism to limit nutrient availability for pathogens, while some pathogens have developed strategies to exploit or manipulate Trp metabolism for their benefit (64); (d) *Immunomodulation and tolerance*: The immunomodulatory effects of Trp catabolism, including the generation of metabolites with immunosuppressive properties, suggest that this pathway has evolved to play a role in regulating the immune response (65). Tolerance induction, mediated by Trp catabolism, could have provided an evolutionary advantage by preventing excessive inflammation and immunopathology, contributing to the host’s ability to coexist with commensal microorganisms; (e) *Nutrient sensing and energy metabolism*: Trp catabolism is not only involved in immune responses but is also linked to broader metabolic processes. The breakdown of Trp can provide precursors for the synthesis of other molecules, and its regulation may be tied to overall energy metabolism.

**Figure 2:**
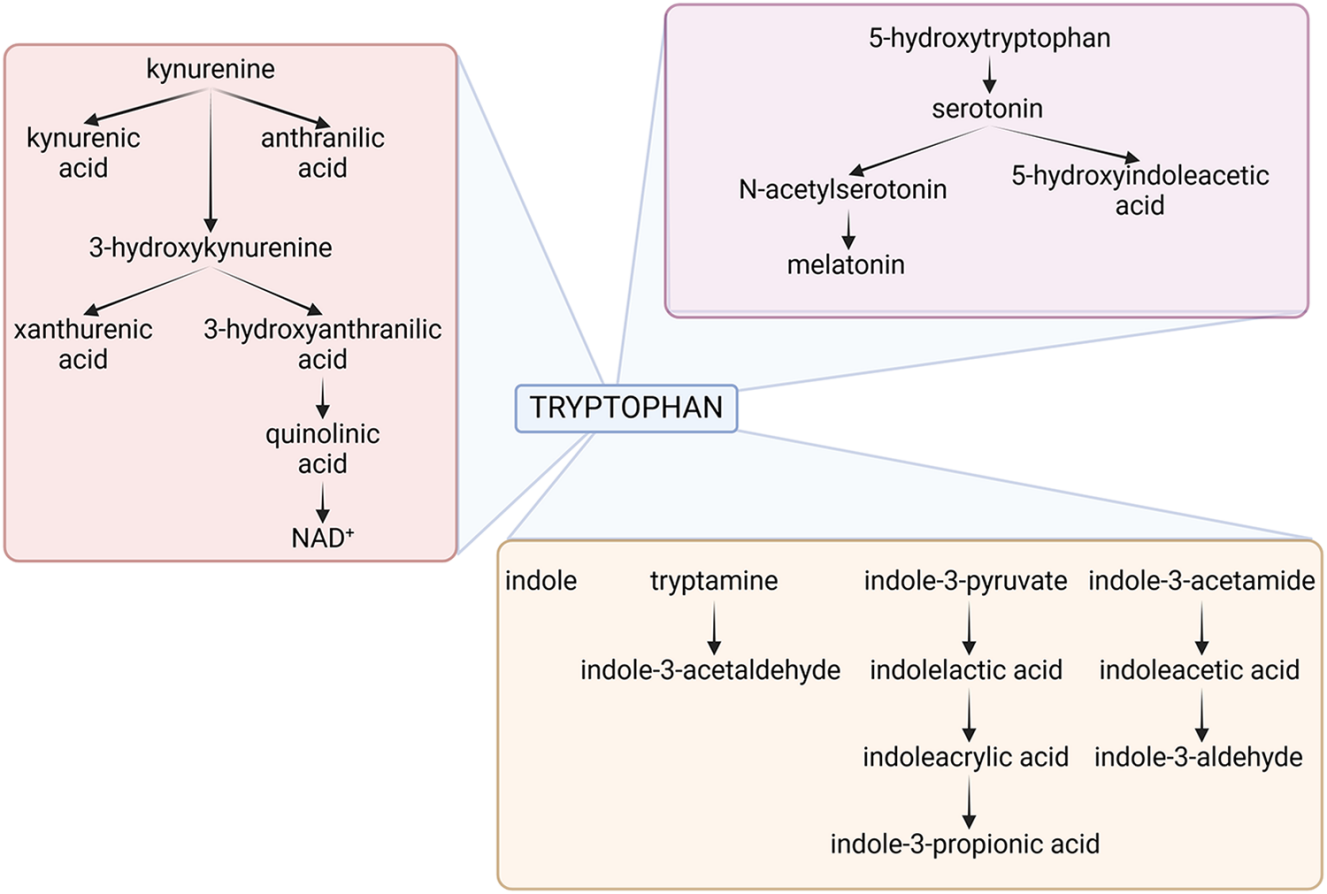
The figure depicts the three main tryptophan metabolic pathways of host and microbial origin, namely the kynurenine (pink box), serotonin (violet box) and indole (brown box) pathways. Details are described in the text. NAD, Nicotinamide Adenine Dinucleotide. Created with BioRender.com.

Therefore, as to the question of why an organism would want to destroy an essential amino acid like Trp, it is important to note that the goal is not necessarily the destruction of Trp but rather the modulation of its levels (63, 66–69). The evolutionary advantage lies in the ability to dynamically regulate Trp availability in response to specific environmental and physiological cues. By controlling Trp levels, organisms can influence their own metabolism, respond to stressors, and shape the host-microbe interaction in ways that promote survival and adaptation. It is interesting to note that bacteria that eat worms use NAD as a ‘food signal’ to open their mouths but, if NAD is unavailable, they stop reproducing and enter a developmental and reproductive arrest phase, mediated by serotonin, to survive (60, 70). In the context of host defense, limiting the availability of essential nutrients like Trp can be a strategic means of thwarting the growth and proliferation of pathogens (Figure 2).

Much like their hosts, bacteria can metabolize Trp along all three pathways, influencing the so-called “brain-gut-microbiota axis”. *Bacteroides* spp., *Clostridium* spp., *Escherichia coli*, *Lactobacillus* spp., *Bifidobacterium* spp., *Akkermansia muciniphila*, *Faecalibacterium prausnitzii*, *Prevotella* spp., *Ruminococcus* spp., all degrade Trp in the gut via the kynurenine pathway (71). There is some evidence that several of the same species may likewise induce serotonin in the gut. *Escherichia coli, Clostridium* Spp., *Bacteroides* spp., *Akkermansia muciniphila, Clostridioides difficile, Enterococcus* spp., *Proteus* spp., *Citrobacter* spp., and *Klebsiella* spp. will instead produce a preponderance of indole or derivatives thereof (72). It follows that—if host and gut bacteria—both metabolize Trp there must be competition for the substrate, yet flexible balance for local and systemic homeostasis (69).

## 4 Tryptophan and the heart

Gut microbiota and microbiota-derived metabolites have been increasingly recognized for their potential impact on CVD, including hypertension, heart failure, myocardial infarction, arrhythmia, atherosclerosis, and myocarditis. Evidence from recent studies has shown that gut microbiota contributes to the development of myocarditis, an inflammatory disease that can result in myocardial damage and the emerging field of cardioimmunology (37, 73, 74). The metabolites produced by gut microbiota can affect the immune system and have an impact on cardiovascular health (10, 40). This is in line with the long-established notion that gut microbiota-dependent metabolites serve as a link connecting the dynamic balance between the host and the gut microbiota (75). In addition to microbial-derived SCFAs, BAs and TMAO, a number of host and microbial metabolites derived from the essential amino acid tryptophan (Trp) degradation has been implicated in cardiovascular diseases (10, 76).

The kynurenine pathway is a metabolic pathway that plays a crucial role in Trp degradation (77–82). This pathway is primarily associated with immune regulation and has been implicated in various physiological and pathological processes, including immune homeostasis and cardiovascular integrity (83). The pathway’s contributions to immune homeostasis include (a) *Regulation of T-cell responses*: The kynurenine pathway is a key player in the modulation of immune responses. One of its main functions is the regulation of T cell activity. Specifically, the production of kynurenine metabolites can influence T cell differentiation and function (84); (b) *Immunosuppressive effects*: Certain metabolites of the kynurenine pathway, such as kynurenine itself, have immunosuppressive properties. They can inhibit the proliferation of T cells and promote the generation of regulatory T cells, which are crucial for maintaining immune tolerance and preventing autoimmune reactions (82). As regards the cardiovascular system, additional functions are: (c) *Regulation of vascular function*: Components of the kynurenine pathway, including kynurenine and its derivatives, have been implicated in vascular function by affecting, for instance, endothelial function and blood vessel integrity (85); (d) *Control of inflammation and atherosclerosis*. The immunomodulatory effects of the kynurenine pathway may influence the inflammatory processes associated with cardiovascular conditions (86–88). As a matter of fact, dysregulation of the kynurenine pathway has been implicated in cardiovascular diseases in which the kynurenine and the [Kyn]/[Trp]-ratio were associated with an increased risk of developing cardiovascular disease (10, 76) while higher Trp levels were associated with a tendency toward lower incident risk of mortality (76).

The serotonin pathway also plays an important role in the degradation of Trp with effects that go beyond its role as neurotransmitter in the central nervous system to include, among others, the regulation the immune and cardiovascular functions. In the periphery, serotonin is a major regulator of vasoreactivity, by directly inducing vasoconstriction in large arteries and veins and exerting a vasodilatory effect in arterioles via nitric oxide release and vascular smooth muscle relaxation (89). Aggregating platelets release serotonin that may contribute to the etiology of spasm in cerebral, digital and coronary vessels, and in the maintenance of the elevated peripheral resistance in arterial hypertension (90). Increased concentrations of serotonin were indeed associated with an increased risk of cardiovascular damage and disease (76, 91).

Finally, the impact of indoles, produced by bacterial degradation of Trp, has gained attention for their dual influence at the pathogen-host interface (84, 92–94). In cardiology, metabolites in the indole pathway did not show consistent associations with cardiovascular outcomes, although higher concentrations of some end products of the indole pathway have been reported to be associated with a lower risk of atherosclerosis (76, 95). For instance, the indole-3-aldehyde (3-IAld) metabolite was found to be lower, different from others, in patients with myocardial infarction (96). The section below delves into the manifold influence of indole metabolites on the host metabolism and immunity, specifically examining their systemic effects from the standpoint of cardioimmunology.

### 4.1 Indoles and systemic immunity

Indole is a heterocyclic organic compound with a bicyclic structure consisting of a six-membered benzene ring fused to a five-membered pyrrole ring. It is found in various natural products such as the amino acid tryptophan and the hormone serotonin. Indole derivatives have been studied in relation to their effects on the cardiovascular system (Box 3).

Box 3 **Key points regarding indole and its derivatives on the cardiovascular system.**

1.⁠ ⁠**Vasodilation**: Some studies have shown that certain indole derivatives exhibit vasodilatory effects, which can help relax blood vessels and lower blood pressure. For example, indole-3-acetic acid has been shown to induce vasodilation in experimental models (97).

2.⁠ ⁠**Anti-inflammatory effects**: Indole derivatives may possess anti-inflammatory properties that can be beneficial in cardiovascular health (98).

3.⁠ ⁠**Antioxidant activity**: Some indole derivatives have been found to have antioxidant activity, which can help reduce oxidative stress in the cardiovascular system. Oxidative stress is implicated in various cardiovascular diseases, and antioxidants can help neutralize harmful free radicals (99).

4.⁠ ⁠**Serotonin receptor modulation**: Indole derivatives play a role in regulating cardiovascular function through the modulation of serotonin receptors.

5.⁠ ⁠**Platelet aggregation**: Some indole derivatives may affect platelet function and aggregation, which are important in the formation of blood clots. Modulation of platelet activity by indole derivatives could have implications for cardiovascular health and thrombotic events (100).

6.⁠ ⁠**Cardioprotective effects**: Certain indole derivatives have been investigated for their potential cardioprotective effects. For example, indole-3-carbinol has been studied for its anti-inflammatory and antioxidant properties, which could contribute to protecting the heart from damage (101, 102).

7.⁠ ⁠**Potential therapeutic applications**: Indole derivatives are being explored for their potential therapeutic applications in CVD such as hypertension, atherosclerosis, and heart failure. Further research is needed to better understand the mechanisms of action and potential benefits of indole derivatives in cardiovascular health (103–105).

### 4.2 Indole and the aryl hydrocarbon receptor: the case of indole-3-aldehyde, 3-IAld

The Aryl hydrocarbon receptor (AhR) is a ligand-activated transcription factor that plays a crucial role in regulating various physiological processes, including immune responses, xenobiotic metabolism, and maintenance of cellular homeostasis (106–108). AhR is a member of the basic helix-loop-helix/Per-ARNT-Sim (bHLH-PAS) protein family and is primarily localized in the cytoplasm in its inactive state. AhR activation occurs when it binds to specific ligands, leading to its translocation into the nucleus, where it forms a complex with its partner, the AhR nuclear translocator. This complex then binds to specific DNA sequences known as xenobiotic response elements in the promoter regions of target genes, thereby regulating their expression.

The gut microbiota has been implicated in the production of AhR ligands (106–110). These ligands are often derived from dietary components and microbial metabolism. Indole derivatives are prominent examples of AhR ligands produced by gut microbiota through the breakdown of dietary tryptophan. Additionally, certain metabolites of tryptophan, such as kynurenine and kynurenic acid, can also activate AhR. The activation of AhR by these microbial-derived ligands has been associated with various immunomodulatory effects. AhR activation in immune cells can regulate the balance between pro-inflammatory and anti-inflammatory responses, impacting the development and function of immune cells. Furthermore, AhR activation in the gut has been linked to the maintenance of intestinal barrier integrity and the regulation of mucosal immune responses (111–113). The interaction between the gut microbiota and AhR highlights the intricate relationship between the microbiome, dietary factors, and host physiology, emphasizing the importance of these interactions in shaping immune and metabolic functions (114). As mentioned above, degradation of Trp by bacteria or enterocytes generates several AhR-binding molecules, mainly serotonin, tryptamine and indoles, including: indole, indole-3-propionic acid (IPA), indole-3-acetic acid (IAA), indole-3-aldehyde (3-IAld), indole-3-acetaldehyde (IAAld), indole-3-lactic acid (ILA), indole acrylic acid and others, including tryptamine and skatole (115). Thus, the co-evolution of microbial communities with mammalian hosts has led to interconnected metabolic pathways that intricately influence both physiological and pathological processes. Trp derivatives, arising from both host and microbial sources, exemplify this metabolic complexity. Among these derivatives, 3-IAld stands out as a metabolite produced by the gut microbiota (92, 116–119).

Initially recognized for its role as an agonist of the AhR, particularly in promoting epithelial barrier functions, this compound has now been implicated in a myriad of activities across various pathological conditions. Along this direction, our group has been involved for some time in turning microbial AhR agonists into therapeutic agents via drug delivery systems (116, 118). By deciphering how signaling molecules, such as 3-IAld, interact with AhR may pave the way for novel therapeutics in inflammatory human diseases, for the realization of which drug delivery platforms are instrumental. So far, three synthetic AhR agonists—laquinimod, tranilast and benvitimod—have been investigated in phase I–III clinical trials. The trials involved patients with autoimmune conditions, such as Crohn’s disease, rheumatoid arthritis, asthma, atopic dermatitis or multiple sclerosis (120). However, to our knowledge, none of them was tested in patients with cardiovascular diseases. A 3-IAld dry powder for inhalation was formulated and assessed for its effectiveness in comparison to oral and intranasal delivery methods in experimental lung inflammation (44). The resulting inhalable dry powder demonstrated: (i) suitability for pulmonary administration, (ii) favorable toxicological safety, and (iii) superior efficacy over alternative administration routes (oral and intranasal) in reducing inflammatory and disease scores. This research advocates for the utilization of 3-IAld inhalable dry powders as a promising and innovative therapeutic approach for targeting inflammation in pulmonary diseases and, likely, cardiopulmonary conditions (121, 122).

In conclusion, AhR plays a crucial role not only in detoxification but also in various physiological processes, particularly in maintaining vascular homeostasis. Despite its high expression in the endothelium, there is a lack of comprehensive understanding of AhR’s function in this context. There is a definite need for consolidating existing knowledge regarding AhR’s involvement in the endothelium and its implications for cardiovascular health (122). Whatever, modulating AhR signaling emerges as a potential therapeutic target for addressing vascular disorders whereby systemic low-grade inflammation reduces circulating endothelial progenitor cells (123). In this regard, it is of interest that while indoxyl sulfate promoted vascular inflammation, IPA and 3-IAld had protective effects (117).

## 5 Future perspectives and conclusions

**Figure 3:**
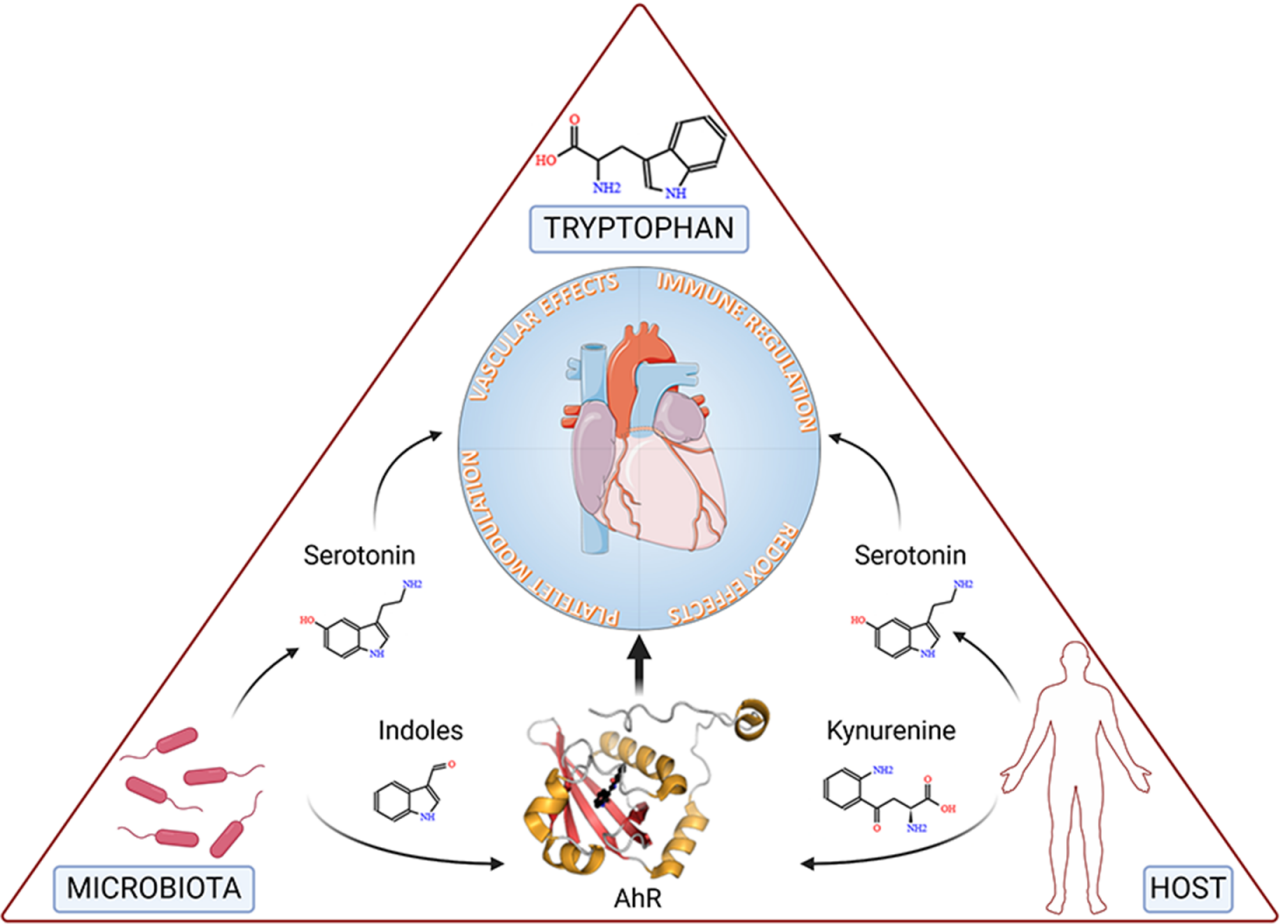
The figure depicts tryptophan, the microbiota and the host at the vertices of a triangle representing the triad involved in cardiovascular health and disease. Specifically, microbial- and host-dependent degradation of tryptophan results in the production of metabolites, including serotonin, indoles and kynurenine, the latter two working as ligands of the Aryl Hydrocarbon Receptor (AhR), ultimately affecting key functions of the cardiovascular system. Details are described in the text. Created with BioRender.com.

Accumulated evidence substantiates the notion that a triad comprising microbiota, Trp, and AhR is a targeted axis in several cardiovascular conditions (Figure 3).

Presently, most treatments primarily address symptoms, focusing on enhancing compromised functionality. However, therapeutic options capable of influencing cellular degeneration or pathology remain elusive. Should a significant role of abnormal intestinal flora composition in specific phenotypes be confirmed, a promising therapeutic avenue could emerge by optimizing pharmacological therapy of CVD with Trp supplementation and/or medications altering the Trp metabolic pathways. Nevertheless, variations among individuals, coupled with the impact of comorbidities, dietary patterns, medications, infections, and lifestyle, can alter gut microbiota composition. Hence, future research demands meticulous investigation using a rigorous experimental framework, taking these factors into account (124). Indeed, despite the potential promise of precisely defined and tailored diets in influencing the microbiome and inflammation, their practical applicability in the general population remains questionable as it is the potential long-lasting benefits of fecal microbiota transplantation in CVD (125).

Additionally, the potential value of the measurement of Trp metabolites as screening and diagnostic tools for a broader population, indicate that mechanistic studies are required to understand the role of Trp metabolism in CVD with the goal to identify new diagnostic and therapeutic options.

Lastly, delving into research on microbiota, Trp catabolic pathways and AhR signaling has uncovered insights into the mechanisms of intestinal distress (126, 127). Despite being underappreciated, a role for the intestinal barrier dysfunction in CVD has become evident in light of its occurrence in hypertension, coronary artery disease, atherosclerosis, heart failure, and myocardial infarction (128). Thus, testing indole derivatives as intestinal barrier-targeted compounds may lead to their potential new class of CVD therapeutics.
